# Salivary apoptotic cells in oral (pre-) cancer as a potential diagnostic means

**DOI:** 10.4317/jced.52212

**Published:** 2015-07-01

**Authors:** Jasdeep Kaur, Constantinus Politis, Reinhilde Jacobs

**Affiliations:** 1 OMFS IMPATH research group, Dept. Imaging & Pathology, Faculty of Medicine, University of Leuven and Oral & Maxillo-facial Surgery, University Hospitals Leuven, Leuven, Belgium

## Abstract

**Background:**

Apoptosis is a genetically programmed form of cell death which is indispensable for development and homeostasis of multi-cellular organism.

**Objectives:**

The aim of this study was to find out the salivary apoptotic cells in oral precancerous and cancerous patients and furthermore to observe the potential diagnostic value of salivary apoptotic cells in detection of oral pre-cancer and cancer.

**Material and Methods:**

Unsimulated saliva was collected from a group of 103 subjects diagnosed with oral (pre-)cancer and a control group of 30 healthy age- and gender-matched individuals. The test group diagnosed with (pre-)cancer was further subdivided in 4 lesion groups oral squamous cell carcinoma (OSCC), oral lichen planus lesions (n=26), oral leukoplakia (n=25), oral sub-mucous fibrosis (n=24) Apoptotic cells were morphologically studied using fluorescence microscopy (TUNEL technique).

**Results:**

While the morphology of apoptotic cells in oral pre-cancer and cancer are morphological similar to the typical epithelial cells of oral cavity mucosa, the number of apoptotic cells was significantly less in OSCC as compared to precancerous and normal healthy tissues.

**Conclusions:**

It could therefore be concluded that salivary apoptotic epithelial cells might be used in early detection and diagnosis of oral pre-cancer and cancer.

** Key words:**Saliva, apoptotic, apoptosis, lichen planus, oral cancer.

## Introduction

Oral cancer is the sixth leading cancer worldwide (1,2). Risk factors such as alcohol and tobacco have an harmonizing effect, other than from infection with human papilloma virus is considered in oral cancer (1). Most of the oral cancer develops from oral premalignant lesions such as leukoplakia, erythroplakia, and lichen planus (3). Oral leukoplakia, submucous fibrosis and lichen planus are major known precursor lesions. The prevalence of malignant transformation of oral lichen planus is around 0.5% and for leukoplakia is around 1% (3). Early detection of oral cancer considerably increases survival rates and diminish other health effects (4). Despite of advancement in technologies, oral cancer cases are diagnosed at very late stage due to lack of awareness of the symptoms and risk factors between public as well as lack of prevention and early detection by oral physicians (5-7). Presently, diagnosis depends mostly on a thorough clinical oral examination and histopathological examination by taking a biopsy. A definite diagnosis is based on biopsy. Various technologies such Chemiluminescence, autofluorescence, Toluidine Blue, Brush biopsy, OralCDx Brush Test, ViziLite Plus with TBlue, VELscope Vx, Sapphire Plus, MicroLux(™) /DL, Identafietc have been proposed, but these technologies need strong evidences to be used in clinical setup (7-8). Chemiluminescence demonstrates good sensitivity for diagnosing any potentially malignant disorders and oral cancer. VELScope cannot precisely differentiate between potentially malignant disorders and erythematous lesions of benign inflammation, which might give false-positive results (7). Early detection is most valuable means to reduce death rate and for good prognosis as well as for better survival rate from diseases (7). It would be beneficial if it could be done through non invasive, cost effective and easy to use technology for early detection of oral pre-cancer and cancer. Such screening could be extremely useful in rural areas with a high incidence of such (pre)cancerous lesions and a very low access to the healthcare system (8).

All normal cells require stimulation to undergo growth, differentiation and proliferations which are specific signals carried by different types of growth factors (9). Angiogenesis is critical in growth, invasion and metastasis of cancer or tumors (9,10). Homeostasis of cell growth and cancer regression is controlled by apoptosis (9) which is further regulated by genes (11). There has been reported disturbance in gene regulation and apoptosis in oral carcinogenesis (12-14). Detection of apoptotic cells in cancer has been identified from different body fluids such as blood, serum and urine. Few studies have been conducted on salivary apoptosis in oral pre-cancer and cancer (14). So, this study was aimed to find out the salivary apoptotic cells in oral pre-cancer and cancer and furthermore, to find the diagnostic value of salivary apoptotic cells in detection of oral cancer and pre-cancer.

## Material and Methods

A total of 103 patients with histopathologically confirmed oral (pre-) cancerous lesions were recruited from Baba Nidhan Singh, Punjab, India for the purpose of this study. Oral leukoplakia lesions, oral lichen planus, oral sub-mucous fibrosis and oral squamous cell carcinoma were identified based on the criteria as proposed by the World Health Organization (13). Matched age and gender normal healthy were selected as control group. Demographic data of patient and control characteristics are shown in  Table 1. The chronic alcohol intoxication ranged from 10 to 120 days {75 (45)} days; and drank an approximately 432 (200-800) g of alcohol/daily. Alcohol-dependent individuals met the criteria for the alcohol and nicotine dependence according to ICD-10 and DSM-IV criteria respectively (15-16). Smokers with average of 18 cigarettes daily were taken as chronic smokers. All selected subjects had no other oral diseases, gingival or periodontal inflammation, nor any oral lesions and symptoms and/ or signs of systematic infections and other diseases. Informed consents were obtained from all subjects. This study was approved by the ethical committee of Baba Nidhan Singh hospital, Punjab, India. Unstimulated whole saliva from subjects was collected over ice. The saliva samples were stored at -4 C for further analysis. The salivary apoptotic cells were detected by using TUNEL assay (Molecular Probes, Inc, Eugene, USA) as described in a previous study (14). Briefly, Saliva cells were incubated for the designated duration before being trypsinized and washed by using phosphate-buffered saline, and fixed in 2% paraformaldehyde for 20 minutes. The cells were washed three times by using phosphate-buffered saline and stored at −20°C in 70% ethanol for 12–18 h prior to performing the TUNEL assay as per manufacturer’s instructions. Total epithelial and apoptotic cells were measured by using bright fields and fluorescent microscope respectively as described as in previous study (14). Inter- and intra observers difference were measured using Pearson correlation by selecting four samples from each group. The examiners were first author (Jasdeep Kaur) and colleague (Balwant Rai). A clinical criterion of examination was number of cells type in fixed area. Each cell type is measured using fraction of particular cell type in percentage of its number in total exfoliated epithelial cells. Pearson co-rrelation between living, apoptotic and dead cells with gender, age, smoking and drinking habits was analyzed. The data were measured using SPSS. 11.0 version (SPSS Inc, Chicago , USA).

**Table 1:**
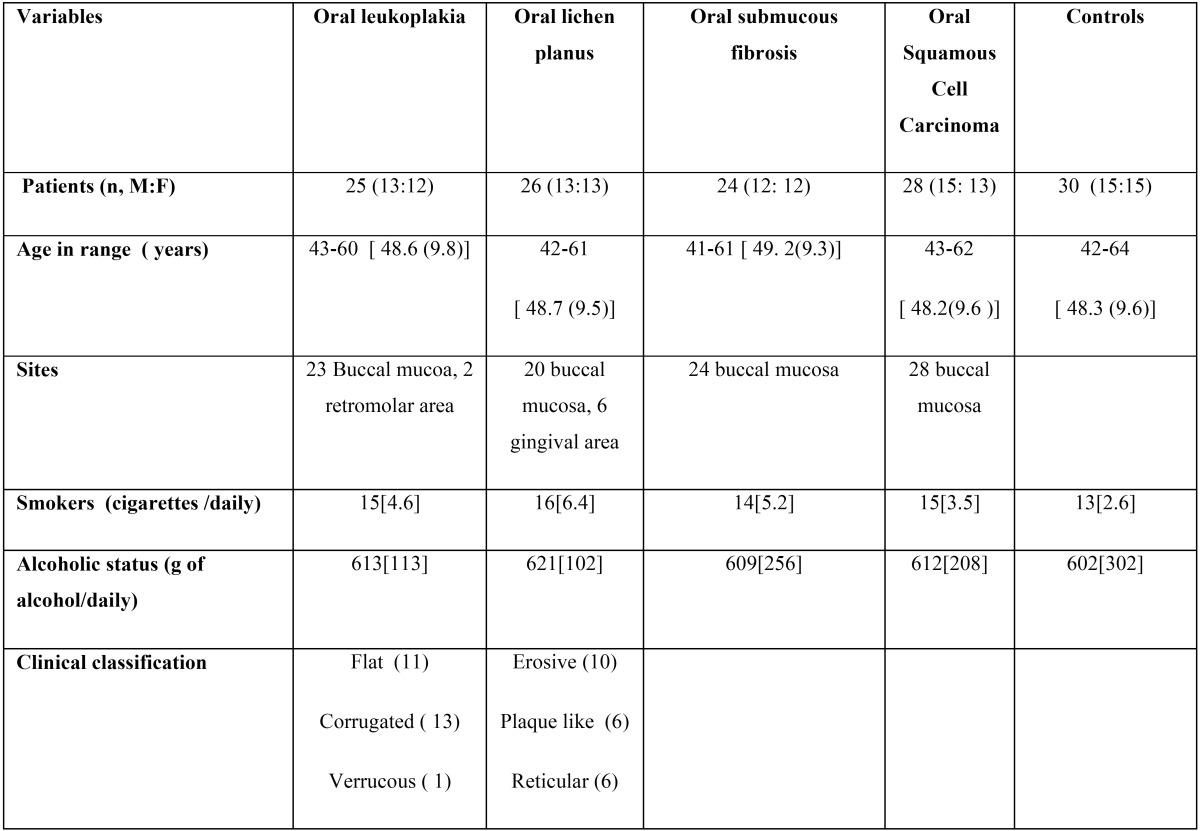
Demographic characteristics of patients and controls.

## Results

The dead, apoptotic and living cells in oral pre-cancer and cancer are morphological similar to the typical epithelial cells of oral cavity mucosa. The living, apoptotic and dead cells are shown in  Table 2 and  Table 3. The Dead cells were significantly higher in SCC as compared to pre-cancerous and normal healthy ( Table 2, Table 3 *p*<0.01), also non-significantly higher in oral precancerous cells as compared to normal healthy. Apoptotic and living cells were significantly lower in SCC as compared to precancerous and normal healthy ( Table 2, Table 3, *p*<0.01). There was also a tendency towards significantly lower values when contrasting oral precancerous tissues to normal healthy oral mucosa. Intra- and interobserver reliability was found to be 95 and 92% respectively (Pearson correlation) ( Table 4, Table 5). We reported non-significant positive correlation between dead & apoptotic cells and smoking and drinking (T Table 6).

**Table 2:**
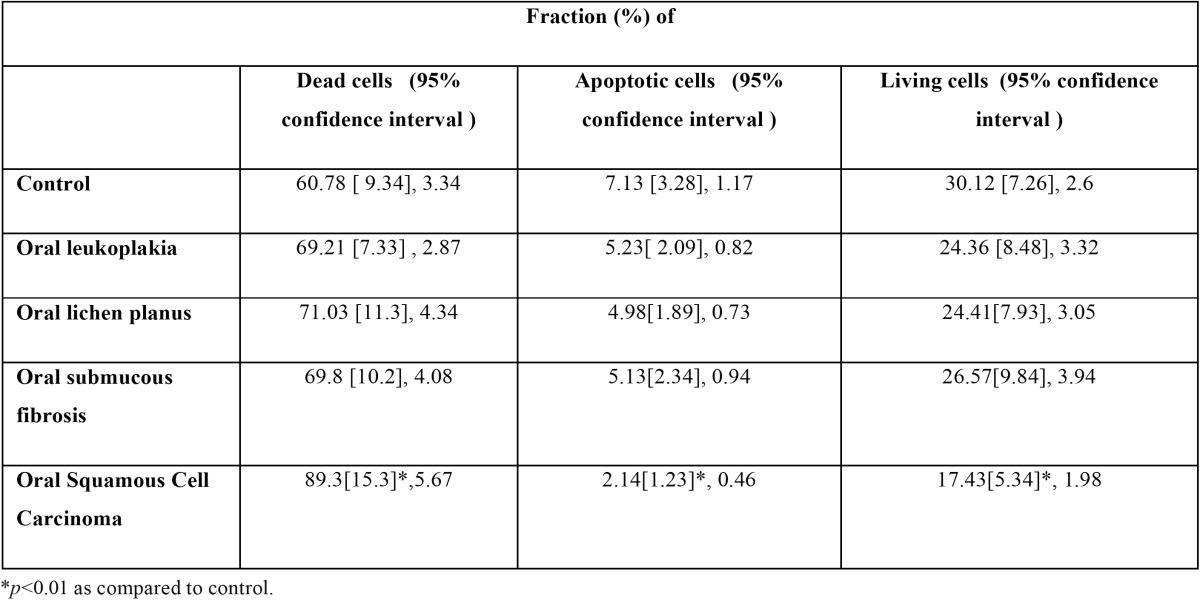
Living, apoptotic and dead cells {Mean [ SD], CI %} in precancerous and cancerous.

**Table 3:**
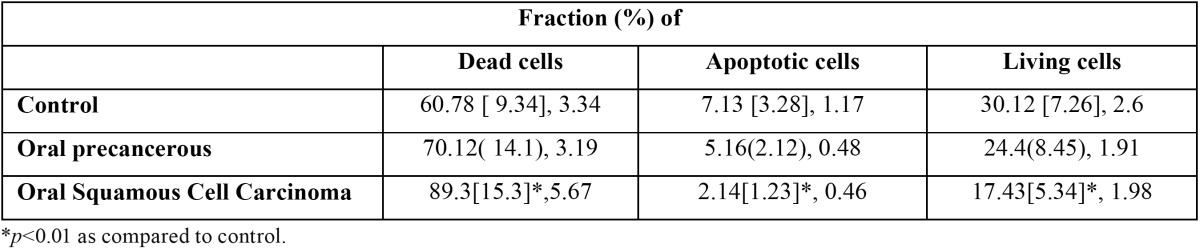
Living, apoptotic and dead cells {Mean [ SD], CI %} in precancerous and cancerous.

**Table 4:**
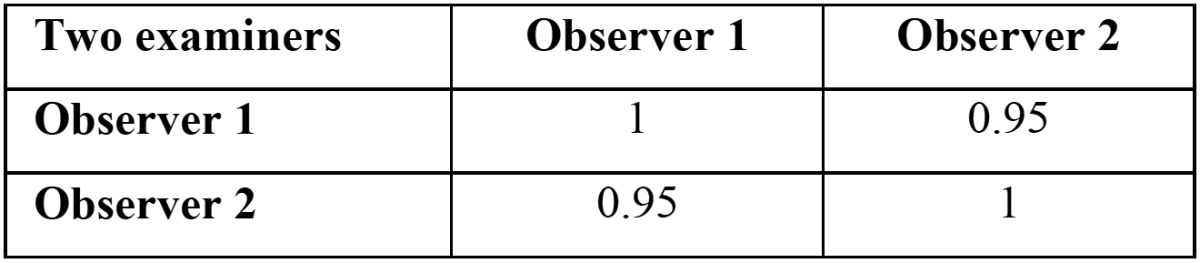
Inter observer correlation ( Cohen kappa).

**Table 5:**
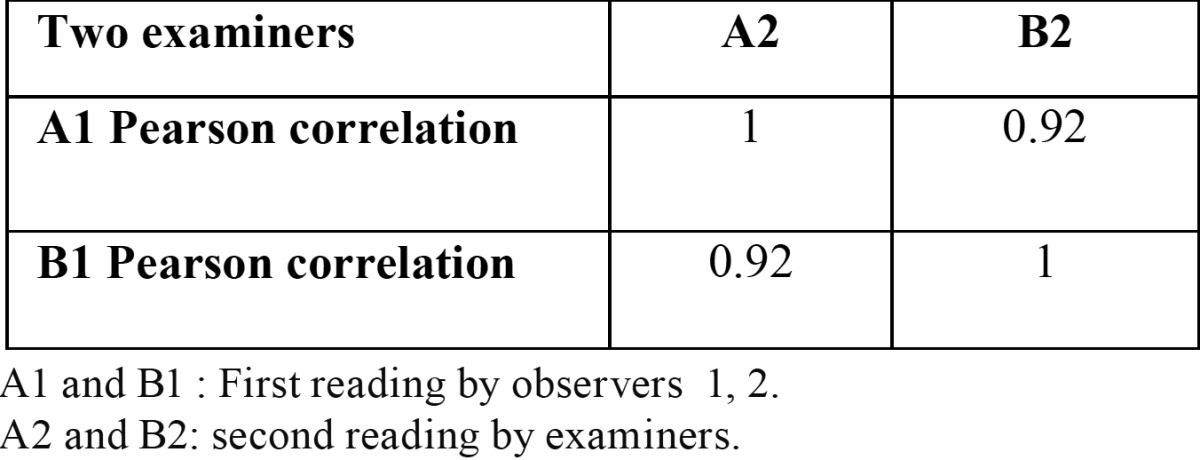
Inter observer correlation (Cohen kappa).

**Table 6:**
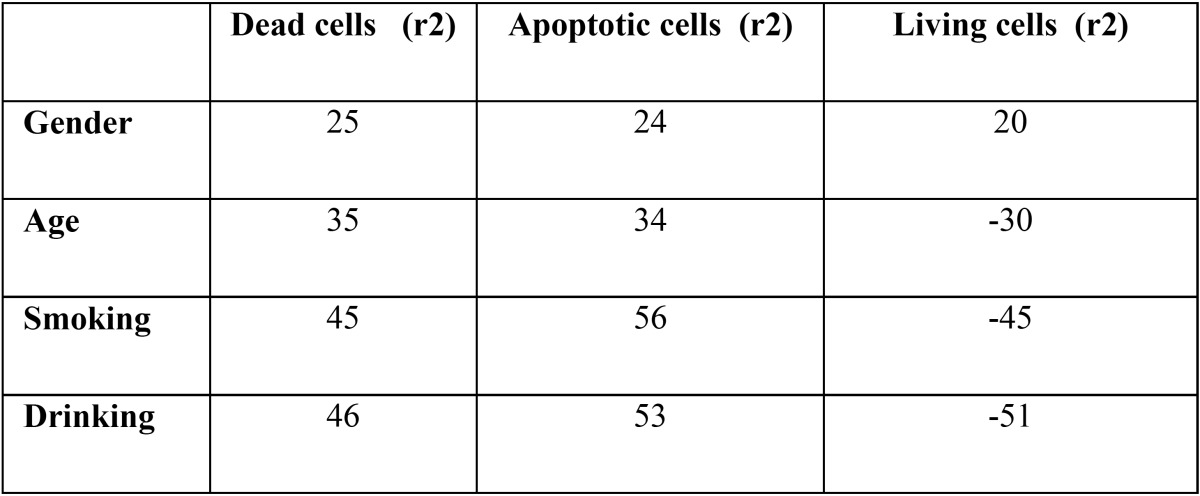
Correlation of Living, apoptotic and dead cells with gender, age, smoking and drinking habits.

## Discussion

Apoptosis is a genetically programmed form of cell death which is vital for development and homeostasis in human (9). Apoptosis is observed in normal healthy individuals as well as in different diseases. Disturbances in apoptotic mechanisms have been associated to an extensive range of pathologies such as oral diseases including oral pre-cancer and cancer (14). Thus, it might be implicated in clinical application for early detection, diagnosis, prognosis and monitoring oral pre-cancer and cancer. Numerous studies have been published on detection of apoptotic cells in pre-cancer and oral cancer using TUNEL (14). The main advantages of this method are less time consumption, high specificity & sensitivity and early detection of apoptosis (14). The inter- and intra-observer reliability was evaluated to be excellent. Our study supports the previous study (14) which shows dead cells are significantly higher in SCC as compared to precancerous and normal healthy, also non-significantly higher in oral precancerous cells as compared to normal healthy. Living cells are significantly lower in SCC as compared to precancerous and normal healthy which supports previous study (14). So, this method can be considered reliable in detection of apoptotic cells. Apoptotic activity is significantly lowered in SCC as compared to pre-cancer and normal healthy which supports previous studies (14,17) while it is contradicted in other studies (18,19). Furthermore, it is non-significantly higher in oral leukoplakia and oral submoucous fibriosis. It might be due to low resistance to apoptosis. The rise in number of neoplastic cells can occur during enhanced proliferation, diminished cell turnover, as well as a combination of proliferation & diminished cell turnover. Tumor cells from extensive types of human cancers have revealed to show increased survival and resistance to apoptosis (20). Down-regulation in cancer cell apoptosis is due to somatic and non-somatic mutation and loss of expression of pro-apoptotic molecules and over-expression of apoptosis inhibitory molecules (21). Many studies have been conducted on saliva based diagnosis of oral cancer and pre-cancer (22-25), but there is very little research published on its cellular components. The dead, apoptotic and living cells in oral pre-cancer and cancer are morphological similar to typical epithelial cells of oral cavity mucosa as supported by a previous study (14). It might be due to inactivate salivary epithelial cells (26). Positive correlation between dead & apoptotic cells and smoking and drinking were found as supported by previous studies (27,28).

Salivary apoptotic cells are significantly less in oral cancer than both precancerous and normal oral tissues. Furthermore, salivary apoptotic cells were non-significantly lower in precancerous than in normal oral tissue. So, salivary apoptotic cells might be potentially useful as a biomarker for distinguishing between cancerous, precancerous and normal healthy oral soft tissues. Further studies are required to find out underlying molecular mechanism between oral epithelium, saliva and apoptosis.

Conclusions: From a clinical point of view, the following conclusions can be drawn:

1) Salivary apoptotic cells might have potential diagnostic and prognostic values as useful biomarkers for detection of cancer from normal healthy with 95% confidence interval.

2) Salivary apoptotic cells might have no prognostic utility in defining those precancerous lesions which will develop into a cancerous lesion.

3) The most valuable contribution of these diagnostic markers could be the use as a screening test in rural areas where a thorough investigation of the oral cavity is impossible and cancerous lesions could remain concealed during a superficial inspection of the oral cavity.

## References

[1] Perez-Sayans M, Somoza-Martin JM, Barros-Angueira F, Reboiras-Lopez MD, Gandara Rey JM, Garcia-Garcia A (2009). Genetic and molecular alterations associated with oral squamous cell cancer (Review). Oncol Rep.

[2] Duvvuri U, Myers JN (2009). (2009) Cancer of the head and neck is the sixth most common cancer worldwide. Curr Probl Surg.

[3] Reibel J (2003). Prognosis of oral premalignant lesions: Significance of clinical, histological, and molecular biological characteristics. Crit Rev Oral Biol Med.

[4] Palmer O, Grannum R (2011). Oral cancer detection. Dent Clin North Am.

[5] Mignogna MD, Fedele S, Lo Russo L, Ruoppo E, Lo Muzio L (2001). Oral and pharyngeal cancer: lack of prevention and early detection by health care providers. Eur J Cancer Prev.

[6] Mashberg A, Samit A (1995). Early diagnosis of asymptomatic oral and oropharyngeal squamous cancers. CA Cancer J Clin.

[7]  Rashid  A,  Warnakulasuriya  S . The use of light-based (optical) detection systems as adjuncts in the detection of oral cancer and oral potentiallymalignant disorders: a systematic review. J Oral Pathol Med.

[8] Scully C, Bagan JV, Hopper C, Epstein JB (2001). Oral cancer: current and future diagnostic techniques. Am J Dent.

[9] Hall P (1991). Cell proliferation. J Pathol.

[10]  Eissa  S,  Shoman  S (1999). Tumor Markers.

[11] Piattelli A, Rubini A, Fiorni M, Iezzi G, Santinelli A (2002). Prevalence of p53, bcl-2, and Ki -67 immunoreactivity and of apoptosis in normal oral epithelium and in premalignant and malignant lesions of the oral cavity. J Oral Maxillofac Surg.

[12] Sidransky D (2002). Emerging molecular markers of cancer. Nat Rev Cancer.

[13] van der Waal I (2009). Potentially malignant disorders of the oral and oropharyngeal mucosa; terminology, classification and present concepts of management. Oral Oncol.

[14] Cheng B, Rhodus NL, Williams B, Griffin RJ (2004). Detection of apoptotic cells in whole saliva of patients with oral premalignant and malignant lesions: a preliminary study. Oral Surg Oral Med Oral Pathol Oral Radiol Endod.

[15] World Health Organization (1999). The ICD-10 Classification of Mental and Behavioural Disorders. World Health Organization.

[16] (1999). Diagnostic and Statistical Manual of Mental Disorders, Fourth Edition. American Psychiatric Association.

[17] Ravi D, Ramadas K, Mathew BS, Nalinakumari KR, Nair MK, Pillai MR (1999). De novo programmed cell dead in oral cancer. Histopathology.

[18] Birchall M, Winterford C, Tripconi L, Gobé G, Harmon B (1996). Apoptosis and mitosis in oral and oropharyngeal epithelia: Evidence for topographical switch in premalignant lesons. Cell Prolif.

[19] Bentz BG, Chandra R, Haines GK 3rd, Robinson AM, Shah P, Radosevich JA (2002). Nitric oxide and apoptosis during human head and neck squamous cell carcinoma development. Am J Otolaryngol.

[20] Kaufmann SH, Gores GJ (2000). Apoptosis in cancer: cause and cure. Bioessays.

[21] Ghavami S, Hashemi M, Ande SR, Yeganeh B, Xiao W, Eshraghi M (2009). Apoptosis and cancer: mutations within caspase genes. J Med Genet.

[22] Rai B, Kaur J, Jacobs R, Singh J (2010). Possible action mechanism for curcumin in pre-cancerous lesions based on serum and salivary markers of oxidative stress. J Oral Sci.

[23] Rai B, Kharb S, Jain R, Anand SC (2007). Salivary vitamins E and C in oral cancer. Redox Rep.

[24] Rai B, Kaur J, Jacobs R, Anand SC (2011). Adenosine deaminase in saliva as a diagnostic marker of squamous cell carcinoma of tongue. Clin Oral Investig.

[25] Yakob M, Fuentes L, Wang MB, Abemayor E, Wong DT (2014). Salivary biomarkers for detection of oral squamous cell carcinoma - current state and recent advances. Curr Oral Health Rep.

[26] Fuchs E, Raghavans S (2002). Getting under the skin of epithelial morphogenesis. Nat Rev Genet.

[27] Nefic H, Handzic I (2013). The effect of age, sex, and lifestyle factors on micronucleus frequency in peripheral blood lymphocytes of the Bosnian population. Mutat Res.

[28] Fenech M, Bonassi S (2011). The effect of age, gender, diet and lifestyle on DNA damage measured using micronucleus frequency in human peripheral blood lymphocytes. Mutagenesis.

